# Experimental and Mechanistic Studies on the Tensile Sensitivity of a PDMS/MWCNT Nanocomposite and Its Application in Concrete Crack Monitoring

**DOI:** 10.3390/ma18050927

**Published:** 2025-02-20

**Authors:** Yongquan Zhang, Weimin Guo, Chengzhe Song, Xinliang Liu, Jinshan Yu, Yong Ge

**Affiliations:** 1 School of Transportation Science and Engineering, Harbin Institute of Technology, Harbin 150090, China; 2 Liaoning Transportation Planning and Design Institute Co., Ltd., Shenyang 110111, China; gwm02401@163.com (W.G.);; 3 Transportation Institute, Inner Mongolia University, Hohhot 010070, China; 4 Inner Mongolia Transportation Design and Research Institute Co., Ltd., Hohhot 010070, China; hityjs@126.com

**Keywords:** PDMS/MWCNT nanocomposite, tensile sensitivity, mechanism of tensile sensitivity, monitoring of concrete cracks, impact load

## Abstract

A polydimethylsilosane/multiwalled carbon nanotube (PDMS/MWCNT) nanocomposite, as a tensile-strain-sensing material, was manufactured using a simple solution casting method. The percolation threshold, the relationship between the temperature and resistance, the tensile sensitivity, and the mechanism of the tensile sensitivity of the PDMS/MWCNT nanocomposite were studied, along with its application in concrete crack monitoring. The results show that the PDMS/MWCNT nanocomposite demonstrated a significant percolation phenomenon. The resistance change ratio of the PDMS/MWCNT nanocomposite changed linearly with the environmental temperature, gradually decreasing with an increasing environmental temperature. The PDMS/MWCNT nanocomposite had a higher tensile sensitivity, and the sensing factor was 6.65 when the volume fraction of carbon nanotubes was 1.26 *v*/*v*% near the percolation threshold, and the sensing factor of the PDMS/MWCNT nanocomposite decreased with an increase in the volume fraction of carbon nanotubes. The relationship between the relative electrical conductivity of the PDMS/MWCNT nanocomposite and the tensile strain can be expressed as ln(*σ*/*σ*_0_) = *Aε*. In addition, the quantitative relationship between the electrical conductivity of the PDMS/MWCNT nanocomposite and the volume fraction of carbon nanotubes was obtained based on the tunneling effect theory and the effective medium model. PDMS/MWCNT nanocomposites can be used as a sensing material to monitor the propagation of concrete cracks under the impact of a free-falling ball.

## 1. Introduction

Research on crack detection and crack width monitoring is always a hot topic. In major structural engineering projects, such as high-rise buildings, large bridges, large sports venues, dams, large oil production platforms, and especially bridges that form important connections in a transportation network, cracks caused by loads and the environment can seriously influence the integrity and safety of structures. Cracks can characterize a change in a structure’s structural bearing capacity and are a crucial signal of structural dangerousness. The appearance of cracks indicates that the interior of concrete structures is damaged and deteriorating. When the cracks extend beyond a certain width (0.2–0.4 mm), this results in more severe steel bar corrosion, concrete carbonization, freezing–thawing erosion, and so on. This secondary damage will seriously impact the normal usage and safety of concrete structures and may ultimately result in their collapse because of the propagation of cracks. Therefore, the crack width is thought to be one of the most important parameters reflecting the extent of damage to a concrete structure. If the crack width is monitored and determined in time, an appropriate plan for reinforcement and maintenance can be worked out, and the service life of concrete structures will be effectively prolonged.

The most used methods in monitoring structural cracks are optical fiber sensors [1,2,3,4,5], image processing [6,7,8,9], piezoelectric sensors [10,11,12], conductive concrete [13,14,15], acoustic emissions [16,17,18], and others [19,20,21]. However, these methods also have some disadvantages. For example, optical fiber sensors are costly, difficult to construct, and easily damaged when repairing concrete structures. Photogrammetry can be used to collect real-time images of structures and has high feasibility for short-term crack monitoring in some small areas, but it is less useful for detecting the initial crack and effectively monitoring cracks in large concrete structures over the long term. Therefore, it is important that flexible sensors with a large tensile strain are used to monitor crack propagation when the width of a crack exceeds the permissible threshold.

A method for monitoring cracks in concrete based on smart films, or flexible sensors, has become a hot research topic in recent years. Bao [22] designed a plastic optical fiber sensing device and demonstrated the relationship between the light intensity loss and crack width under different fiber/crack angles. Yue [23] employed a tapered polymer fiber sensor to detect a crack in a concrete beam, and the experimental results also indicated that TPFSs can be used for post-crack detection. Zhang’s group developed several smart films, namely silver slurry arrays that adhere to fragile resin film [24], an enameled copper wire neural network with and without plastic film [25], and a capacitive sensor with parallel enameled copper wire film [26]. Ghaz [27] developed silver-based conductive paint, as sensing skin was shown to be able to detect cracking in concrete structures, though it could not be used to monitor the crack width. Glisic [28] detected the occurrence and growth of cracks through observing strain changes using a large sensing sheet based on large-area electronics. Schumacher [29] developed a continuous conductive skin consisting of carbon nanotubes, which was sensitive to changes in the strain and the formation and propagation of micro- and macro-damage. Another smart skin [30,31] and smart paint [32] were used to monitor cracks, having been developed through digital image correlation and machine learning, respectively. This method showed a relative advantage in detecting crack occurrences and monitoring crack growth. However, the problem of crack width monitoring with smart films has not yet been solved.

This study developed a tensile-strain-sensing material consisting of polydimethysilosane as the polymer matrix and multiwalled carbon nanotubes as the conductive filler, prepared using a solution casting method. The percolation of the PDMS-MWCNT nanocomposite samples, the electrical resistance at different temperatures, the tensile sensitivity when subjected to large tensile strains (ε = 50%), and the mechanism of tensile sensitivity were all characterized. In addition, a dynamic impact load test was used to further develop this flexible tensile strain material to detect and monitor initial visible cracks and the propagation of concrete cracks.

## 2. Materials and Methods

### 2.1. Experimental Materials and Mix Proportions

Polydimethylsilosane (PDMS) and multiwalled carbon nanotubes (MWCNTs) were purchased from Dow Corning (Sylgard 184, USA) and Timesnano Inc. (TNGM2, China), respectively (outer diameter: 10–20 nm; length: 10–30 μm; and electrical conductivity: >100 S/m). The volume fractions of MWCNTs added to the PDMS matrix were 0.21 *v*/*v*%, 0.42 *v*/*v*%, 0.84 *v*/*v*%, 1.26 *v*/*v*%, 1.68 *v*/*v*%, 2.10 *v*/*v*%, 2.52 *v*/*v*%, 3.36 *v*/*v*%, and 4.20 *v*/*v*%. P·O 42.5 Ordinary Portland cement was used as the cementitious material in this experiment. The fine aggregate was natural river sand with a fineness modulus of 2.9, and the coarse aggregate was continuous grading crushed stones with sizes ranging from 5 mm to 20 mm. A polycarboxylate-based super plasticizer was selected to enhance the workability of fresh concrete. The two different polypropylene fibers used in this study were (i) fine polypropylene fiber (length: 8–32 mm; diameter: 0.05 mm) and (ii) thick polypropylene fiber (length: 18 mm; diameter: 0.60 mm), and twelve concrete mix proportions were designed for the experiment, as listed in Table 1.

The PDMS/MWCNT nanocomposite was prepared using a solution casting method [33]. Firstly, the polydimethylsilosane base polymer was added into a tetrahydrofuran solution to keep the solution concentration at 1.0 g/mL and magnetically stirred for 30 min at a speed of 1000 rpm. Multiwalled carbon nanotubes were added into another tetrahydrofuran solution to keep the solution concentration at 20 mg/mL and dispersed with ultrasonic stirring for 2 h at a speed of 1000 rpm. Secondly, the two above-mentioned solutions were poured together into a container, subjected to further ultrasonic vibrating, and magnetically stirred for 2 h at a speed of 1000 rpm. Thirdly, the obtained mixed solution of the PDMS base, MWCNTs, and tetrahydrofuran was placed on a hot plate at a temperature of 80 °C to evaporate the tetrahydrofuran while the solution was magnetically stirred at 600 rpm. Fourthly, when the tetrahydrofuran was completely evaporated, a PDMS curing agent (a mass ratio of 10:1 compared to the PDMS base) was added into the mixture of the PDMS base and MWCNTs, and it was further magnetically stirred for 10 min at 100 rpm or mechanically stirred for 10 min at room temperature. At last, the mixture obtained from the previous step was cast into a stainless-steel mold, put into a vacuum-drying oven for 1 h at 80 °C, and then further cured in an oven for 2 h at 150 °C. An elastomer nanocomposite was finally prepared. The obtained nanocomposite was cut into 55 mm × 5 mm × 1 mm specimens for testing.

A laboratory-type mixer with a lateral axis and a 60 L capacity was used to manufacture the concrete. First, the aggregates and cement were poured into the mixer and mixed for 1 min. Second, the polypropylene (PP) fiber was added into the aggregate–cement mixture in the dry phase, and a mixing process was performed for 2 min until a nearly homogeneous mixture was obtained. Finally, water was added into the mixture along with the plasticizer, and the wet mixture was homogeneously mixed for 4 min. This process was accomplished in the same way throughout the preparation of all the concrete mixtures. In this experiment, five prism specimens in each group with a size of 100 mm × 100 mm × 400 mm were manufactured. After following a 28-day standard curing process, the specimens were ready for testing.

### 2.2. Test Methods

In order to achieve repeatable results, all the experiments were conducted in a stable laboratory environment controlled using climatic test chambers unless stated otherwise. The resistance of the PDMS/MWCNT nanocomposite specimen was recorded in real time using a UT805A digital multimeter (Uni-Trend Group Limited, China) with a measurement range of 1 × 10^−5^ Ω to 20 MΩ. The percolation phenomenon of the PDMS/MWCNT nanocomposite was calculated from sheet resistance measurements using a four-point probe. In the following experiments, copper sheets were adhered to two ends of the PDMS/MWCNT nanocomposite specimen using a conductive silver paste as electrodes to form a two-probe measurement setup. PDMS/MWCNT nanocomposites with different volume fractions above the threshold were chosen, and the sensitivity properties were studied. The temperature resistance results were obtained in the range of −25 °C to 80 °C, and the temperature was controlled using a refrigerator and heat oven. The tensile strain resistance results were obtained with the help of a CSS-88000 series electronic universal testing machine. The electrical conductivity of the PDMS/MWCNT nanocomposite was calculated using Equation (1).

(1) $$\sigma =\frac{l}{RV_{0}}=\frac{l_{0}^{2}{\left(1+\varepsilon \right)}^{2}}{RV_{0}}$$

where σ, $l_{0}$, ε, R, and $V_{0}$ are the electrical conductivity, initial length, tensile strain, resistance, and volume of the PDMS/MWCNT nanocomposite, respectively, assuming $V_{0}$ is invariable during the stretching process.

**Figure 1:**
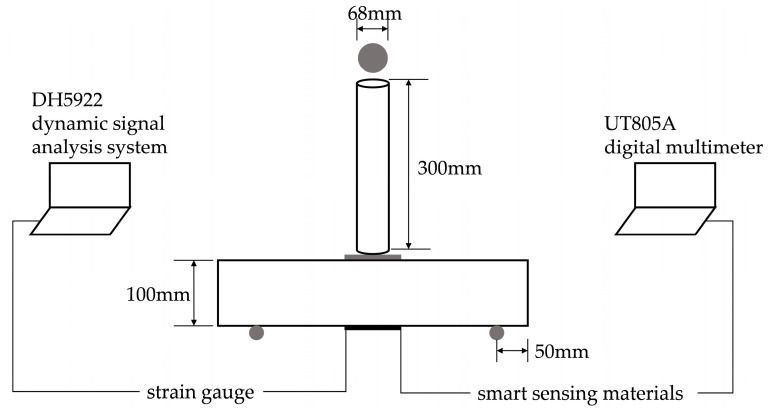
Schematic illustration of monitoring of PP fiber concrete crack under dynamic impact load test.

The test system displayed in Figure 1 was modified from [34] to make it suitable for monitoring cracks in PP fiber concrete. The impact testing device works according to the principle that the steel ball falls freely onto the specimens to produce a certain amount of impact energy. The weight and diameter of the steel ball were 1.25 kg and 68 mm, respectively; the height of the free fall was 300 mm; the span of the PP fiber concrete specimen’s bottom was 300 mm; and a steel model with a size of 100 mm × 100 mm × 5 mm was placed into the upper center area of the PP fiber concrete specimen. The strain gauge and PDMS/MWCNT nanocomposite sample were glued parallel to each other with resin in the bottom center area of the PP fiber concrete specimen. The value of the strain gauge was monitored in real time using the DH5922 dynamic test system. The resistance of the PDMS/MWCNT nanocomposite was recorded in real time using a UT805A digital multimeter (Uni-Trend Group Limited, China), and the width of the crack in the bottom surface of the PP fiber concrete specimen was measured using a ZBL-F800 crack test instrument (ZBL SCI & TECH, China). There were five specimens in each PP fiber concrete group. In this experiment, the strain gauge served as a reference to determine whether visible cracks had occurred on the bottom surface of the PP fiber concrete specimen. When the DH5922 dynamic signal analysis system detected that the resistance of the strain gauge showed the maximum absolute value after a certain number of free-falling ball impacts, it determined that the strain gauge was damaged, and it checked whether visible cracks had appeared on the bottom surface of the PP fiber concrete specimens. When a visible crack occurred, before the next free-falling ball impact, time was allowed to measure the width of the crack in the PP fiber concrete specimen. The impact tests continued until the PP fiber concrete specimen fractured.

## 3. Results and Discussions

### 3.1. Percolation Threshold

**Figure 2:**
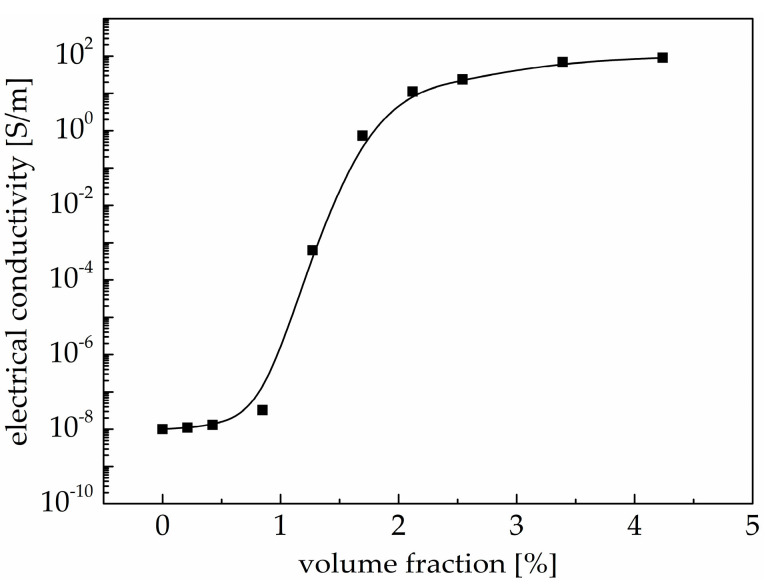
The electrical conductivity of PDMS/MWCNT nanocomposites with different volume fractions of carbon nanotubes.

As can be seen in Figure 2, the electrical conductivity of PDMS/MWCNT nanocomposites increased with an increase in the MWCNT volume fraction; that is, the conductive properties of the nanocomposites gradually improved. The electrical conductivity of the PDMS/MWCNT nanocomposites demonstrated a steep increase when the volume fraction of MWCNTs was larger than 0.84 *v*/*v*%, suddenly increasing by several orders of magnitude. The electrical conductivity of the nanocomposites increased slowly and tended to be stable, remaining at the same order of magnitude when the volume fraction of the MWCNTs was larger than 3.36 *v*/*v*%. Therefore, the electrical conductivity of PDMS/MWCNT nanocomposites with varying volume fractions of carbon nanotubes had an abrupt change inflection point, which is the largest tangent slope of the percolation curve. The volume fraction of carbon nanotubes corresponding to this abrupt change inflection point was 1.06 *v*/*v*%, achieved through differential treatment of the percolation curve. The volume fraction, the most critical component of carbon nanotubes, is also known as the percolation threshold, and this is the percolation phenomenon. There was no fully connected conductive path in the PDMS/MWCNT nanocomposites when the volume fraction of carbon nanotubes was less than 1.06 *v*/*v*% or the distance between two adjacent carbon nanotubes was bigger than the minimum distance of the tunneling conductive effect. As a result, the PDMS/MWCNT nanocomposites had an insulation property similar to that of the PDMS matrix. A fully connected conductive path began to form in the PDMS/MWCNT nanocomposites when the volume fraction of carbon nanotubes was larger than 1.06 *v*/*v*%, and this resulted in a steep increase in electrical conductivity. More conductive paths formed and the distance between two adjacent carbon nanotubes decreased with an increase in the volume fraction of carbon nanotubes, so the number of conductive paths gradually became stable after reaching a certain number. The slow increase in the electrical conductivity of the PDMS/MWCNT nanocomposites was the most intuitionistic reflection [35].

### 3.2. Effect of Temperature on Resistance

**Figure 3:**
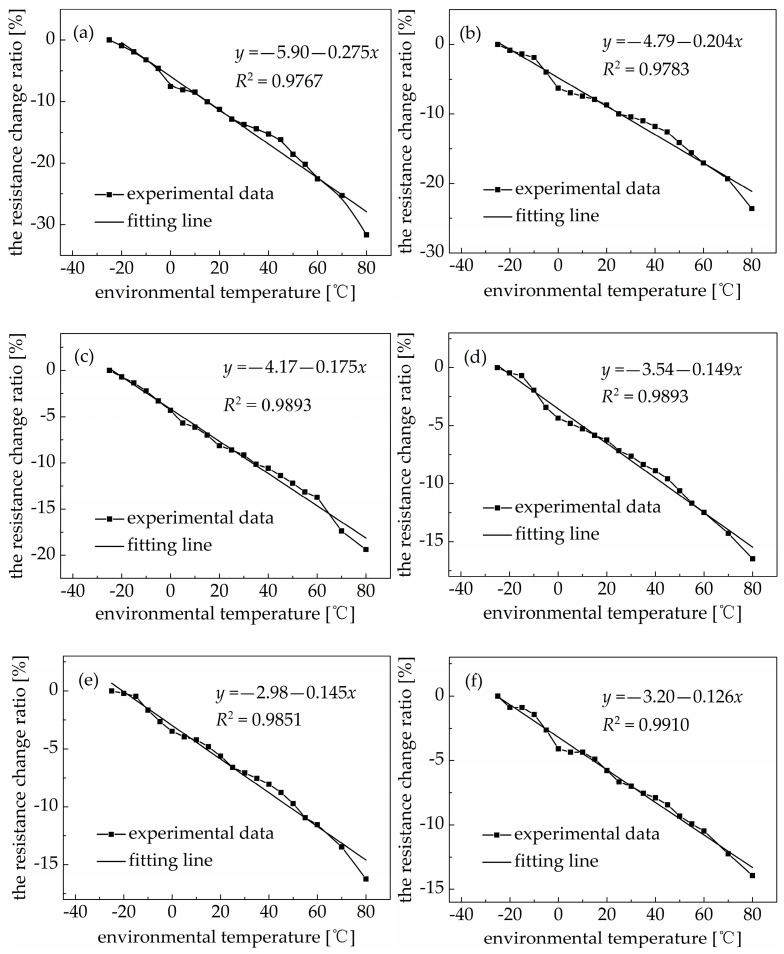
The resistance change ratio of PDMS/MWCNT nanocomposites with different volume fractions of carbon nanotubes under different environmental temperatures: (**a**) 1.26 *v*/*v*%; (**b**) 1.68 *v*/*v*%; (**c**) 2.10 *v*/*v*%; (**d**) 2.52 *v*/*v*%; (**e**) 3.36 *v*/*v*%; (**f**) 4.20 *v*/*v*%.

The effect of the environmental temperature on the resistance of a PDMS/MWCNT nanocomposite is an important factor that must be considered when it is used as a sensing material in practical applications for concrete structures. Figure 3 shows the effect of the environmental temperature on the resistance of PDMS/MWCNT nanocomposites with 1.26 *v*/*v*%, 1.68 *v*/*v*%, 2.10 *v*/*v*%, 2.52 *v*/*v*%, 3.36 *v*/*v*%, and 4.20 *v*/*v*% of carbon nanotubes. The absolute value of the resistance change ratio of the PDMS/MWCNT nanocomposites increased linearly when the environmental temperature rose from negative twenty degrees Celsius to as high as eighty degrees Celsius. In other words, the resistance of the PDMS/MWCNT nanocomposites decreased linearly with an increase in the environmental temperature. The PDMS matrix is an elastomer material, so the volume of the PDMS matrix remained nearly unchanged when the temperature changed. The effect of the environmental temperature on the electrical conductivity of the PDMS/MWCNT nanocomposites was mainly influenced by the transfer velocity and transition of electrons.

The effect of the environmental temperature on the resistance of the PDMS/MWCNT nanocomposites gradually decreased with an increase in the volume fraction of carbon nanotubes. A smaller falling range of the resistance and a smaller slope of the fitting line were the most direct phenomena observed. When the environmental temperature rose from negative twenty degrees Celsius to as high as eighty degrees Celsius, the resistance of PDMS/MWCNT nanocomposites with 1.26 *v*/*v*%, 1.68 *v*/*v*%, 2.10 *v*/*v*%, 2.52 *v*/*v*%, 3.36 *v*/*v*%, and 4.20 *v*/*v*% of carbon nanotubes decreased by 31.64%, 23.62%, 19.38%, 16.48%, 16.22%, and 13.93%, respectively. When the volume fraction of carbon nanotubes increased, the conductive path in the nanocomposite increased, and the distance between two adjacent carbon nanotubes decreased, so the effect of the environmental temperature was weaker. This also illustrates that the resistance of a PDMS/MWCNT nanocomposite undergoes a bigger change when the volume fraction of carbon nanotubes is near the percolation threshold. Further, a differential circuitry configuration could be applied to offset the effect of the environmental temperature on the change in the resistance of the PDMS/MWCNT nanocomposites.

### 3.3. Tensile Sensitivity

**Figure 4:**
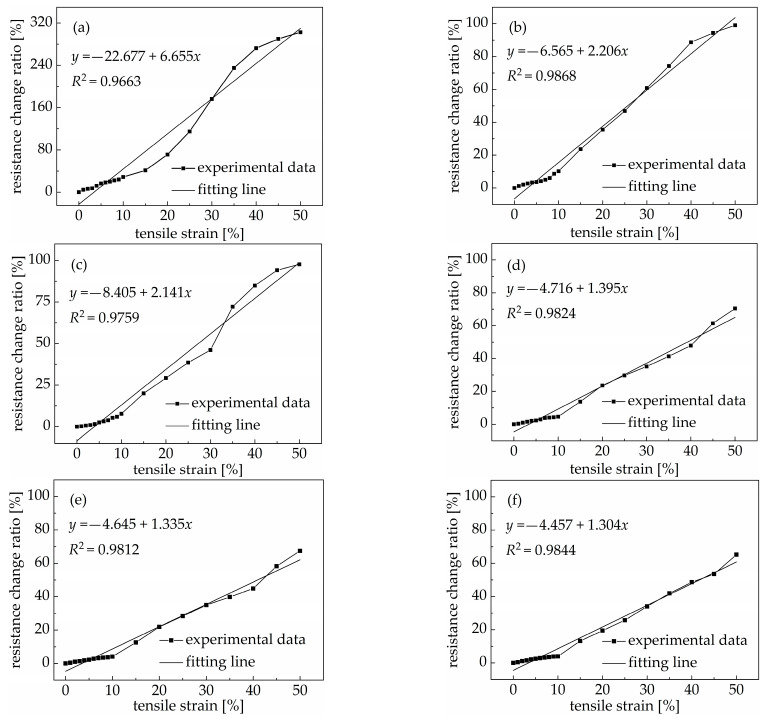
The resistance change ratio of PDMS/MWCNT nanocomposites with different volume fractions of carbon nanotubes under different tensile strains: (**a**) 1.26 *v*/*v*%; (**b**) 1.68 *v*/*v*%; (**c**) 2.10 *v*/*v*%; (**d**) 2.52 *v*/*v*%; (**e**) 3.36 *v*/*v*%; (**f**) 4.20 *v*/*v*%.

The tensile sensitivity is a sensitive property of PDMS/MWCNT nanocomposites’ conductivity that responds to changes in the tensile stress or tensile strain and can be expressed by the relationship between the resistance change ratio and tensile strain of the nanocomposite. In this section, we describe an experiment conducted mainly to study the relationship between the resistance change ratio and tensile strain of nanocomposites with different volume fractions under tensile loading. In order to better explain the tensile sensitivity, the relationship between the resistance change ratio and tensile strain of nanocomposites with different volume fractions of carbon nanotubes under tensile loading was investigated. As can be seen in Figure 4, the resistance of the PDMS/MWCNT nanocomposites increased with an increase in the tensile strain; that means the electrical conductivity decreased with an increase in the tensile strain. When the tensile strain rose from 0 to 50%, the resistance of PDMS/MWCNT nanocomposites with 1.26 *v*/*v*%, 1.68 *v*/*v*%, 2.10 *v*/*v*%, 2.52 *v*/*v*%, 3.36 *v*/*v*%, and 4.20 *v*/*v*% of carbon nanotubes increased by 302.7%, 99.0%, 97.7%, 70.4%, 67.4%, and 65.1%, respectively. When the volume fraction of carbon nanotubes was near the percolation threshold, the distance between two adjacent carbon nanotubes in the nanocomposite was larger, and the tunneling conductive effect was dominant. The distance between two adjacent carbon nanotubes increased when the nanocomposite was stretched, and this damaged the existing conductive path. Moreover, because of the significant difference in the elastic modulus between the PDMS matrix and carbon nanotubes, their deformation differs under the same tensile strain. Thus, shear stress occurred between the PDMS matrix and carbon nanotubes, and the interfacial zone was damaged [36].

In this study, the sensing factor (SF) of the PDMS/MWCNT nanocomposites was calculated using Equation (2).

(2) $$SF=\frac{\Delta R/R_{0}}{\Delta l}$$

where ΔR is the resistance change value of the PDMS/MWCNT nanocomposite, $R_{0}$ is the initial resistance value of the PDMS/MWCNT nanocomposite, and Δl is the change value of the tensile strain.

The smart sensing materials used in structural health monitoring rely on a fast and regular electric response under external load conditions. As such, the sensitivity factor is a good index. The sensitivity factors of PDMS/MWCNT nanocomposites with different MWCNT volume fractions were obtained under different ranges of the tensile strain by inputting the experimental data into Equation (2). As shown in Figure 4, when the tensile strain was in the range of 0 to 50%, the sensitivity factors of nanocomposites with 1.26 *v*/*v*%, 1.68 *v*/*v*%, 2.10 *v*/*v*%, 2.52 *v*/*v*%, 3.36 *v*/*v*%, and 4.20 *v*/*v*% of MWCNTs were 6.65, 2.20, 2.14, 1.39, 1.33, and 1.30, respectively. These results show that the PDMS/MWCNT nanocomposites had good tensile sensitivity when the volume fraction of carbon nanotubes was near the percolation threshold, and the sensing factor decreased with an increase in the volume fraction of carbon nanotubes. This was mainly the coeffect of the conductive path and tunneling conductive effect, with the tunneling conductive effect playing a dominant role. The elastic modulus of the PDMS/MWCNT nanocomposites increased with the volume fraction of carbon nanotubes, so the deformation of the nanocomposites decreased at the same tensile strain. Additionally, carbon nanotubes overlapped with each other in the PDMS matrix, and as the distance between two adjacent carbon nanotubes decreased, the conductive path increased, and the relative change in the electrical conductivity of the PDMS/MWCNT nanocomposites was not significant at a certain tensile strain.

### 3.4. Mechanism of Tensile Sensitivity

The main basis of the conductivity theory considered in this study was the tunneling effect theory, so an effective medium model was used to explain the conductive percolation phenomenon of PDMS/MWCNT nanocomposites, and a tensile sensitivity model was established by combining the theoretical and experimental results. The electrical conductivity of the PDMS/MWCNT nanocomposites prepared in this study fell in the range of 10^−8^ to 90 S/m, so this nanocomposite is defined as a semiconductor.

The microscopic electrical conductivity of conductive materials is expressed by Equation (3).

(3) σ=nqu

where σ is the electrical conductivity of conductive materials, n is the carrier number of the unit volume, and q is the charge quantity of each carrier.

Otherwise, the carrier number of the unit volume semiconductor is calculated using Equation (4).

(4) $$n=N\exp(-\frac{\omega }{kT})$$

where ω is the energy activated by a single electron or a single hole. When the electric field intensity is constant, ω is a constant value. N is a constant related to the material, and k is the Boltzmann constant.

The carrier of the PDMS/MWCNT nanocomposite can transfer into the carbon nanotubes and traverse the interfacial zone from one carbon nanotube to another adjacent carbon nanotube. The electron transfer velocity in carbon nanotubes is nearly identical to the velocity of light and does not change with the conductor. However, it is difficult for electrons to traverse the interfacial zone, and the transfer velocity is largely limited, even though the distance between two adjacent carbon nanotubes is small (100 nm). Therefore, the width of the interfacial zone determines the carrier transfer velocity. When the volume fraction of the conductive filler is in the range of the percolation threshold, the electrical conductivity of the conductive nanocomposite can be expressed by Equation (5) based on the tunneling conductivity theory [37].

(5) $$\sigma =nq\frac{3\sqrt{2m\varphi }}{2\alpha }{\left(\frac{q}{h}\right)}^{2}E\exp(-\frac{4\pi \alpha }{h}\sqrt{2m\varphi })$$

where m is the quality of a single electron, α is the distance between two adjacent carbon nanotubes, φ is the potential barrier between two adjacent carbon nanotubes, h is the Planck constant, and E is the electric field intensity.

Tensile strain is induced in the conductive nanomaterials by tensile loading, because the elastic modulus of PDMS is greatly different from that of carbon nanotubes. Thus, the tensile strain of the nanotubes can be ignored: the tensile strain of the conductive nanomaterials is the same as that of PDMS. Applying tensile strain to PDMS results in a change in the distance between two adjacent carbon nanotubes, and the electrical conductivity of the conductive nanocomposites changes. When the conductive nanocomposite is stretched, the relative electrical conductivity of the conductive nanocomposite is expressed by Equation (6).

(6) $$\frac{\sigma }{\sigma_{0}}={\left(1+\varepsilon \right)}^{-1}\exp(-\frac{4\pi }{h}\sqrt{2m\varphi }\alpha_{0}\varepsilon )$$

where *ε* is the tensile strain of the conductive nanocomposite, $\sigma_{0}$ is the initial resistivity of the conductive nanocomposite, $\alpha_{0}$ is the distance between two adjacent carbon nanotubes before tensile loading is applied, and $\frac{4\pi }{h}\sqrt{2m\varphi }$ is constant under a certain volume fraction of MWCNTs.

Equation (6) can be expressed by Equation (7) through mathematical transformation. When *ε* is very small, Equation (7) can be expressed by Equation (8).

(7) $$\ln\frac{\sigma }{\sigma_{0}}=-\ln(1+\varepsilon )-\gamma \alpha_{0}\varepsilon$$

(8) $$\ln\frac{\sigma }{\sigma_{0}}=-(1+\gamma \alpha_{0})\varepsilon$$

**Figure 5:**
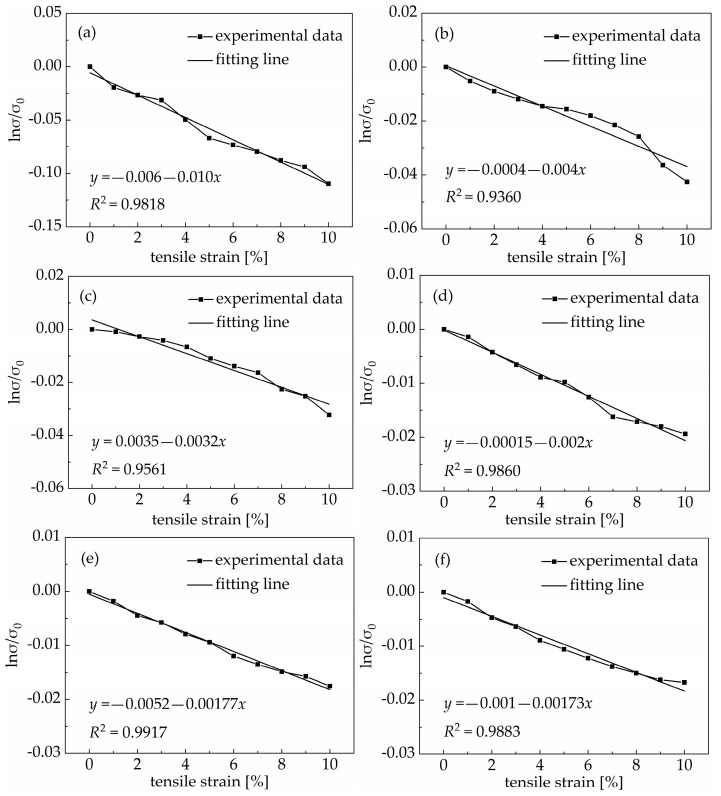
Relationship between electrical conductivity and tensile strain of PDMS/MWCNT nanocomposites with different volume fractions of carbon nanotubes: (**a**) 1.26 *v*/*v*%; (**b**) 1.68 *v*/*v*%; (**c**) 2.10 *v*/*v*%; (**d**) 2.52 *v*/*v*%; (**e**) 3.36 *v*/*v*%; (**f**) 4.20 *v*/*v*%.

This experiment studied the relationship between the electrical conductivity and the tensile strain of PDMS/MWCNT nanocomposites with different volume fractions of multiwalled carbon nanotubes, ranging from 1.26 *v*/*v*% to 4.20 *v*/*v*%, and verified the reasonability of Equation (8) under 0 to 10% tensile strain in the PDMS/MWCNT nanocomposites. The experimental results are shown in Figure 5. In Figure 5, it can be seen that the relationship between the electrical conductivity and the tensile strain of PDMS/MWCNT nanocomposites with different volume fractions under 0 to 10% tensile strain agrees with Equation (8). The electrical conductivity of the PDMS/MWCNT nanocomposites under any tensile strain in the range of 0 to 10% can be calculated using Equation (8). Otherwise, the absolute value of the fitting line slope gradually decreased with an increase in the volume fraction of multiwalled carbon nanotubes. This phenomenon also illustrates that the sensing factor gradually decreased with an increase in the volume fraction of multiwalled carbon nanotubes, but the difference was not significant.

A PDMS/MWCNT nanocomposite is a typical discontinuous 1–3-type composite material, so the orientation of the carbon nanotubes largely influences the change in its electrical conductivity with an increase in the multiwalled carbon nanotube volume fraction. In order to better consider the orientation of carbon nanotubes in a PDMS matrix, this study established a tensile sensitivity model based on experimental results for an effective medium model. The conductive property of PDMS/MWCNT nanocomposites with a certain volume fraction of carbon nanotubes showed a non-linear conductive percolation phenomenon, according to the percolation threshold found in this research. The Bruggeman universal effective medium model (9) can be used to express the non-linear conductive percolation phenomenon [38].

(9) $$\frac{\left(1-\psi )(\sigma_{l}^{1/t}-\sigma^{1/t}\right)}{\sigma_{l}^{1/t}-A\sigma^{1/t}}+\frac{\psi (\sigma_{h}^{1/t}-\sigma^{1/t})}{\sigma_{h}^{1/t}-A\sigma^{1/t}}=0$$

where A is equal to $(1-\psi_{c})/\psi_{c}$, ψ is the volume fraction of carbon nanotubes, $\psi_{c}$ is the percolation threshold, σ is the electrical conductivity of the nanocomposite, $\sigma_{h}$ is the electrical conductivity of the carbon nanotubes, $\sigma_{l}$ is the electrical conductivity of the PDMS matrix, and t is a parameter related to the type of conductive filler [39].

The PDMS matrix is an insulator, so its electrical conductivity can be ignored compared with that of the carbon nanotubes; that is, $\sigma_{l}$→0, and when ψ is larger than $\psi_{c}$, Equation (9) can be expressed by Equation (10). Equation (10) can be expressed by Equation (11) through mathematical transformation. Because the conductive property of carbon nanotubes depends on their structure, the electrical conductivity of the carbon nanotubes is a constant value.

(10) $$\sigma =\sigma_{h}{\left(\frac{\psi -\psi_{c}}{1-\psi_{c}}\right)}^{t}$$

(11) $$\mathrm{lg}\sigma =\mathrm{lg}\sigma_{h}+t\mathrm{lg}(\frac{\psi -\psi_{c}}{1-\psi_{c}})$$

**Figure 6:**
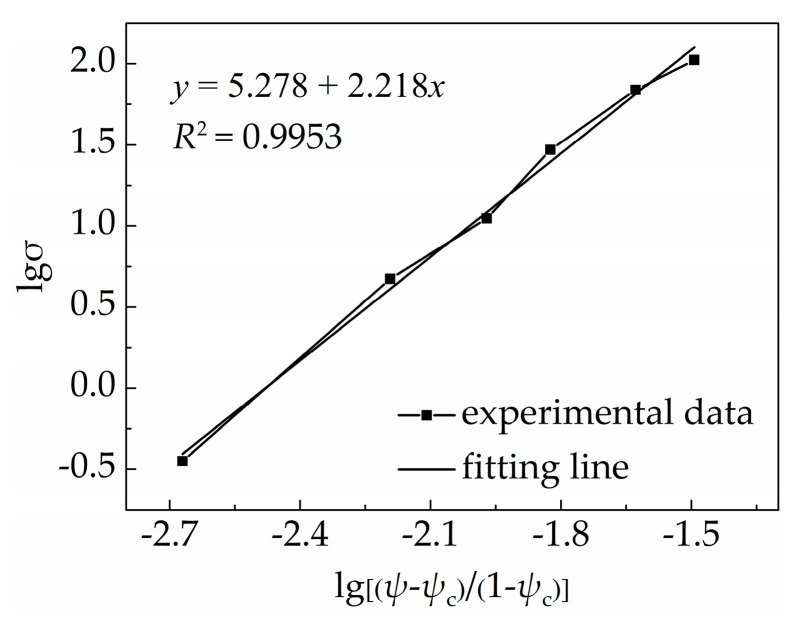
Fitting relationship between electrical conductivity of PDMS/MWCNT nanocomposite and volume fraction of carbon nanotubes.

The percolation threshold of the PDMS/MWCNT nanocomposite was 1.06 *v*/*v*% in this experiment. lgσ and $\mathrm{lg}(\frac{\psi -\psi_{c}}{1-\psi_{c}})$ were defined as a dependent variable and an independent variable, respectively, according to Equation (11). Then, the relative experimental data were put into Equation (11), and Figure 6 shows the final calculation results.

In Figure 6, the linear fitting relationship between lgσ and $\mathrm{lg}(\frac{\psi -\psi_{c}}{1-\psi_{c}})$ is expressed as y=5.278+2.218x. The square of the correlation coefficient is 0.9953 and the slope of the fitting line is 2.218; that is, the parameter t is related to the type of conductive filler. The relationship between the electrical conductivity of the PDMS/MWCNT nanocomposites and the volume fraction of multiwalled carbon nanotubes is expressed by Equation (12) combined with the above-mentioned fitting results and Equation (11). The larger the value of parameter t, the more obvious the percolation phenomenon of the PDMS/MWCNT nanocomposite [40,41]. However, the value of the percolation threshold is also closely related to the dispersion state of the conductive filler, in addition to the polymer matrix and its crystalline degree. If the carbon nanotubes display an agglomeration phenomenon in the PDMS matrix, the percolation threshold of the PDMS/MWCNT nanocomposite is larger because of the decreasing conductive paths as a result of this phenomenon. In addition, a crimp, a knot, and a random orientation of carbon nanotubes occurred in the process of stirring the nanocomposite, so the effective length diameter ratio of the carbon nanotubes decreased, and this also resulted in the percolation threshold increasing.

(12) $$\sigma =1.9\times 10^{5}{\left(1.0107\psi -0.0107\right)}^{2.218}$$

**Figure 7:**
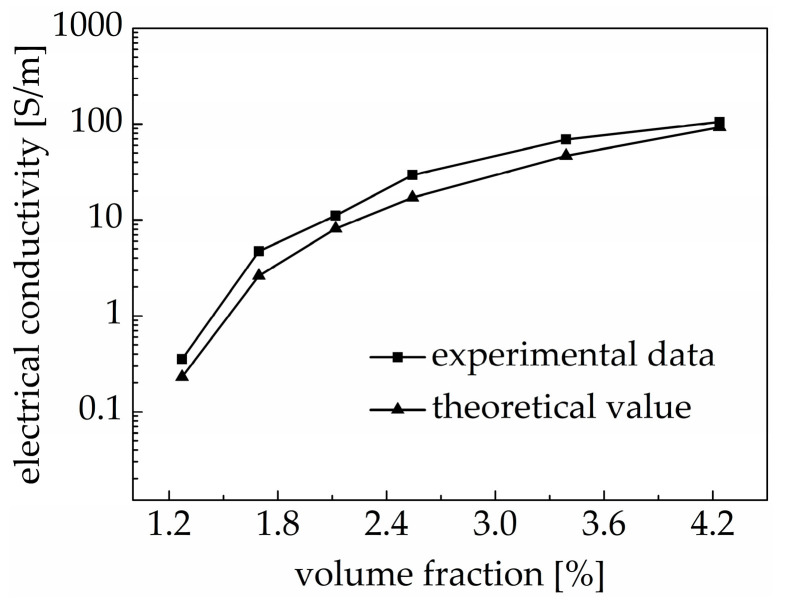
Theoretical value and experimental value of electrical conductivity of PDMS/MWCNT nanocomposites with different volume fractions of carbon nanotubes.

As can be seen in Figure 7, the experimental value was larger than the theoretical value calculated using Equation (12), but the difference is small. These results show that the effective medium model better illustrates the conductive percolation phenomenon of the PDMS/MWCNT nanocomposites.

### 3.5. Detection and Monitoring of PP Fiber Concrete Cracks

**Figure 8:**
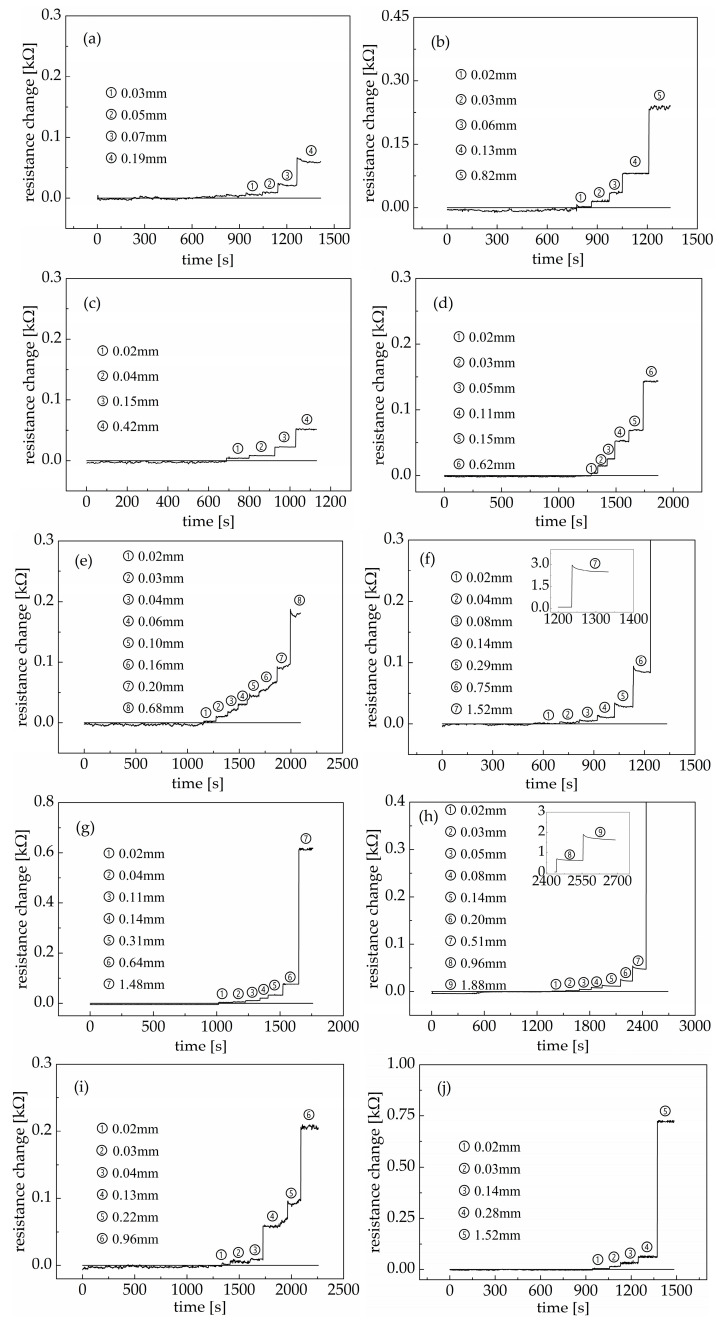

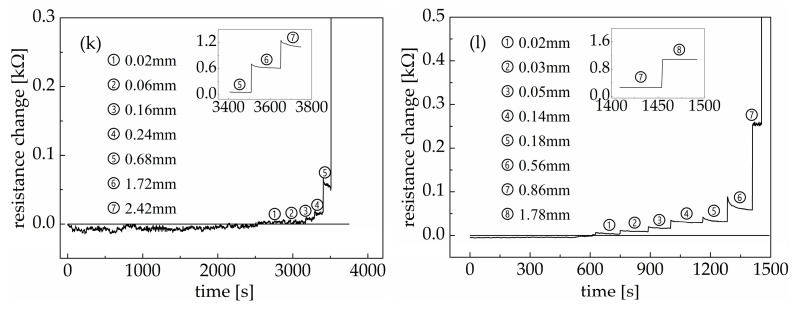
Resistance change of the PDMS/MWCNT nanocomposite with the propagation of PP concrete cracks over time under a free-falling ball impact: (**a**) F1; (**b**) F2; (**c**) F3; (**d**) F4; (**e**) T1; (**f**) T2; (**g**) T3; (**h**) T4; (**i**) FT1; (**j**) FT2; (**k**) FT3; (**l**) FT4.

In order to verify the tensile sensitivity of the PDMS/MWCNT nanocomposites, a nanocomposite with 2.10 *v*/*v*% of carbon nanotubes was selected to monitor cracks in polypropylene-fiber-reinforced concrete in this study, since a nanocomposite at this concentration yields relatively high conductivity and good tensile sensitivity. As shown in Figure 8, the resistance of the nanocomposite remained nearly unchanged or showed minimal changes compared to the initial resistance of the nanocomposite before the occurrence of a visible crack (0.01 mm−0.02 mm) on the bottom of the PP fiber concrete specimen. When a visible crack occurred, the resistance of the nanocomposite increased with an increase in the width of the crack in the bottom of the PP fiber concrete specimen. In addition, the resistance of the nanocomposite rapidly increased when the PP fiber concrete specimen was subjected to a free-falling ball impact. This indicates that the width of the crack in the concrete increased, and the results of the crack test verified this phenomenon. In addition, the change in the resistance of the nanocomposite sometimes fluctuated to a small extent before the next free-falling ball impact. This was mainly because, when measuring the width of cracks on the bottom surface of polypropylene fiber concrete specimens caused by free-falling ball impacts, the cracks will open or close to a certain extent. However, this phenomenon did not affect the subsequent test of the crack width owing to its strict and careful operation.

**Figure 9:**
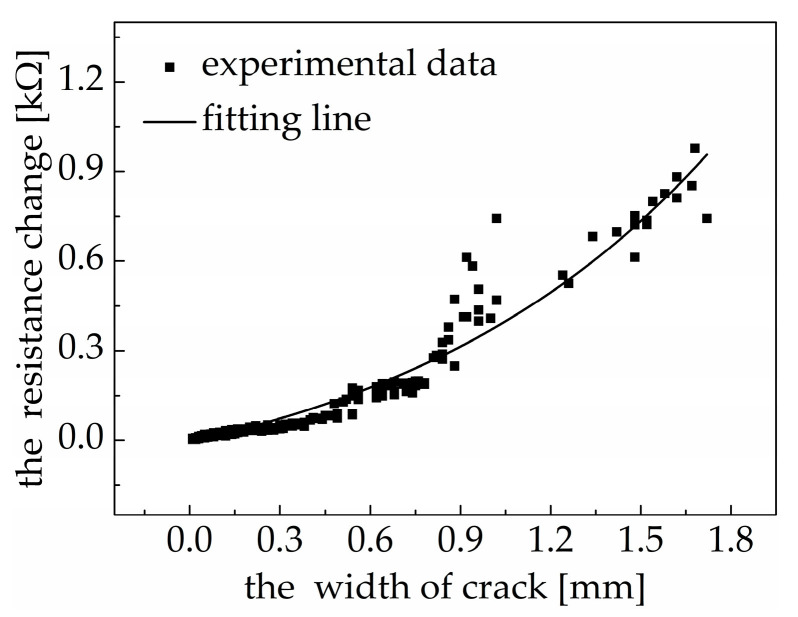
Relationship between the resistance change of the PDMS/MWCNT nanocomposites and the width of the crack in the PP fiber concrete.

When the crack width is 0.33 mm (internal exposure) and 0.41 mm (external exposure), the bearing capacity of reinforced concrete structures will deteriorate [42]. Therefore, this paper divides concrete cracks into two grades, 0.02~0.41 mm and >0.41 mm, which are defined as a safe grade and a damaged grade, respectively. Cracks will not have a negative effect on the integrity and service performance of concrete structures when the crack width is at a safe grade, while cracks will have a negative effect on the integrity and service performance of concrete structures when the crack width is at a damaged grade. This negative effect will be intensified with an increase in the crack width. As shown in Figure 9, when the width of the crack in the bottom surface of the polypropylene fiber concrete specimen was 0.02 mm, the resistance change value of the PDMS/MWCNT nanocomposite was in the range of 0.002~0.008 kΩ. Although the change value was not significant, the occurrence of initial visible cracks in the concrete could be detected using the resistance change value of the PDMS/MWCNT nanocomposite by strictly controlling the test conditions (the environmental temperature was mainly kept constant) and amplifying the signal. When the width of the crack in the bottom surface of the polypropylene fiber concrete specimen was less than 0.41 mm, the resistance change value of the PDMS/MWCNT nanocomposite did not exceed 0.068 kΩ. This experimental result indicates that when the resistance change value of a PDMS/MWCNT nanocomposite is less than 0.068 kΩ, it can be considered that the concrete crack width is within the safe range, and the stability of concrete structures can be maintained without repair. However, when the crack width develops to this extent, if the concrete structure is subjected to a sudden larger external load, the crack width will dramatically increase to the unsafe range, so it is necessary to closely monitor the propagation of the concrete crack width or take an effective maintenance measure. When the width of the crack in the bottom surface of the polypropylene fiber concrete specimen was larger than 0.80 mm, the resistance change values of the PDMS/MWCNT nanocomposites were all greater than 0.200 kΩ. At this time, the width of the crack in the bottom surface of the polypropylene fiber concrete specimen was in the damaged range, so timely and effective reinforcement measures would have needed to be adopted to ensure the safety of the concrete structure.

As shown in Figure 9, the width of the crack in the polypropylene fiber concrete and the resistance change value of the PDMS/MWCNT nanocomposites have a good exponential relationship. The exponential function is expressed by Equation (13), and the goodness of fit is 0.9456.

(13) $$\Delta R=-0.25523+0.04551\exp(\frac{\delta +1.84491}{1.08623})$$

where ΔR is the resistance change value of the PDMS/MWCNT nanocomposite, and δ is the width of the crack in the PP fiber concrete.

The fitting result shows that the monitoring of the concrete crack width can be realized based on the resistance change value of PDMS/MWCNT nanocomposites, and the width of concrete cracks can be accurately calculated using Equation (13). PDMS/MWCNT nanocomposites, when used as a sensor material, can not only be used to detect the occurrence of initial concrete cracks but also to monitor the propagation of the crack width. Piezoceramic smart aggregates integrated with concrete structures can be used to make the real-time monitoring of structural cracks happen but are not sufficient to monitor the crack propagation [43,44]. The monitoring range of an optical fiber Bragg grating sensor is 0.02~0.5 mm, which can be accurately evaluated using the spectrum width [45]. There is a new mechanochromic sensor that can effectively evaluate the concrete crack width up to 1.70 mm when combined with visible image analysis [46]. This demonstrates that flexible sensors have better potential to monitor increases in the crack width of concrete.

## 4. Conclusions

This paper describes our experimental and mechanistic studies on the tensile sensitivity of PDMS/MWCNT nanocomposites manufactured using a simple solution casting method. PDMS/MWCNT nanocomposites, as a sensor material, were also used to monitor the cracks in PP fiber concrete under dynamic impact loads. The conclusions of this study can be drawn as follows:

(1) PDMS/MWCNT nanocomposites showed an obvious percolation phenomenon, with a threshold of 1.06 *v*/*v*%. The resistance of PDMS/MWCNT nanocomposites with different volume fractions of carbon nanotubes showed a nearly linear change in the environmental temperature range of −25 °C to 80 °C and deceased with an increase in the environmental temperature. However, the effect of the environmental temperature on the resistance of PDMS/MWCNT nanocomposites reduced with an increase in the volume fraction of carbon nanotubes. PDMS/MWCNT nanocomposites showed good tensile sensitivity under a uniaxial tensile load, and the relationship between the resistance change and tensile strain of PDMS/MWCNT nanocomposites was approximately linear in the tensile strain range of 0~50%. The sensitivity of PDMS/MWCNT nanocomposites decreased with an increase in the volume fraction of carbon nanotubes, and the highest sensing factor of the PDMS/MWCNT nanocomposites was 6.65 when the volume fraction of carbon nanotubes was near the threshold (1.26 *v*/*v*%).

(2) The effective medium model is suitable to be used in the study of the tensile sensitivity mechanism of PDMS/MWCNT nanocomposites. The relationship between the relative electrical conductivity and tensile strain of PDMS/MWCNT nanocomposites is an exponential function under lower tensile strain, which can be expressed by lg(*σ*/*σ*_0_) = *Aε*. Additionally, the absolute value of coefficient A decreases with an increase in the volume fraction of carbon nanotubes, which verifies the rule of a change occurring in the electrical conductivity and tensile sensitivity of PDMS/MWCNT nanocomposites with a change in the volume fraction of carbon nanotubes. The effective medium model better illustrates the percolation phenomenon and tensile sensitivity of PDMS/MWCNT nanocomposites, and the relationship between the electrical conductivity of PDMS/MWCNT nanocomposites and the volume fraction of carbon nanotubes was obtained by the fitting of experimental results combined with the tunneling effect theory and the effective medium model. The equation used to calculate this was $\sigma =1.9\times 10^{5}{\left(1.0107\psi -0.0107\right)}^{2.218}$.

(3) The resistance of the PDMS/MWCNT nanocomposites synchronously changed with the width of the crack in the polypropylene fiber concrete before the complete fracturing of the PDMS/MWCNT nanocomposite and PP fiber concrete under a free-falling ball impact. When the width of the crack in the bottom surface of the polypropylene fiber concrete specimen was 0.02 mm, the resistance change value of the PDMS/MWCNT nanocomposite fell in the range of 0.002 kΩ~0.008 kΩ. When the width of the crack in the bottom surface of the polypropylene fiber concrete specimen was less than 0.41 mm, the resistance change value of the PDMS/MWCNT nanocomposite did not exceed 0.068 kΩ. When the width of the crack in the bottom surface of the polypropylene fiber concrete specimen was larger than 0.80 mm, the resistance change values of the PDMS/MWCNT nanocomposite were greater than 0.200 kΩ. The change in the resistance of the PDMS/MWCNT nanocomposites and the width of the cracks in the PP fiber concrete showed a good exponential relationship within the crack width range of 0.02 mm to 1.72 mm. The predicting equation was $\Delta R=-0.25523+0.04551\exp(\frac{\delta +1.84491}{1.08623})$. Therefore, PDMS/MWCNT nanocomposites can be used as good sensing materials for monitoring the occurrence and propagation of concrete cracks under free-falling ball impacts.

The flexible sensing material had a good linear relationship with the environment, the tensile sensitivity mechanism could be illustrated using the effective medium model, and the resistivity change showed a good mathematical relationship with the concrete crack width; these results demonstrate the suitability of PDMS/MWCNT nanocomposites as a sensing material in a crack deformation evaluation equation according to cracks in fiber-reinforced concrete. This study lacked the determination of the location, shape, and depth of concrete cracks; we plan to conduct further research on these issues using a sensor array or a combination of other proven techniques (i.e., visible images, acoustic emissions, and so on).

## Figures and Tables

**Table 1:** The mix proportions of the PP-fiber-reinforced concrete.

Cement [kg/m^3^]	Water [kg/m^3^]	Fly Ash [kg/m^3^]	Sand [kg/m^3^]	Gravel [kg/m^3^]	Plasticizer [kg/m^3^]	PP Fiber i [kg/m^3^]	PP Fiber ii [kg/m^3^]	PP Fiber i + ii [kg/m^3^]
344	194	86	685	1072	10.1	0.4, 0.8, 1.2, 1.6	—	—
344	194	86	685	1072	10.1	—	1.5, 3.0, 4.5, 6.0	—
344	194	86	685	1072	10.1	—	—	0.8 + 1.5, 0.8 + 3.0 0.8 + 4.5, 0.8 + 6.0

## Data Availability

The original contributions presented in this study are included in the article. Further inquiries can be directed to the corresponding author.
